# Highly efficient neuromorphic learning system of spiking neural network with multi-compartment leaky integrate-and-fire neurons

**DOI:** 10.3389/fnins.2022.929644

**Published:** 2022-09-28

**Authors:** Tian Gao, Bin Deng, Jiang Wang, Guosheng Yi

**Affiliations:** School of Electrical and Information Engineering, Tianjin University, Tianjin, China

**Keywords:** spiking neural network, multi-compartment LIF, neuromorphic system, dendrite-guided synaptic plasticity, on-chip learning system

## Abstract

A spiking neural network (SNN) is considered a high-performance learning system that matches the digital circuits and presents higher efficiency due to the architecture and computation of spiking neurons. While implementing a SNN on a field-programmable gate array (FPGA), the gradient back-propagation through layers consumes a surprising number of resources. In this paper, we aim to realize an efficient architecture of SNN on the FPGA to reduce resource and power consumption. The multi-compartment leaky integrate-and-fire (MLIF) model is used to convert spike trains to the plateau potential in dendrites. We accumulate the potential in the apical dendrite during the training period. The average of this accumulative result is the dendritic plateau potential and is used to guide the updates of synaptic weights. Based on this architecture, the SNN is implemented on FPGA efficiently. In the implementation of a neuromorphic learning system, the shift multiplier (shift MUL) module and piecewise linear (PWL) algorithm are used to replace multipliers and complex nonlinear functions to match the digital circuits. The neuromorphic learning system is constructed with resources on FPGA without dataflow between on-chip and off-chip memories. Our neuromorphic learning system performs with higher resource utilization and power efficiency than previous on-chip learning systems.

## Introduction

A spiking neural network (SNN) is considered the brain-inspired mechanism in computing. It has shown excellent efficiency in many fields. Compared with the huge acquisition of computation resources and memory bandwidth in artificial neural networks (ANNs), SNN provides a convenient way of integrating storage and computing, which contributes to high efficiency (Mead, 1990; Schuman et al., 2017; Davies et al., 2018; Bohnstingl et al., 2019). ANNs focus on continuous functions and gradient descent learning rules. SNNs learn through synaptic plasticity based on the sparse spikes of neurons. The synaptic weights in an SNN are updated based on local errors instead of global gradients backpropagated through layers, which is considered the key to neuromorphic hardware (Boybat et al., 2018; Stewart et al., 2020). Neuromorphic hardware systems have become the core of hardware acceleration and embedded systems (Vivancos et al., 2021).

However, two factors limit efficiency in computation and power. One is the high-throughout data transmission between off-chip and on-chip memory. Although high-performance memory systems have been proposed to optimize data transmission, the power consumption is irreducible (Lian et al., 2019; Vivancos et al., 2021). Dynamic random-access memory (DRAM) is usually used as the off-chip memory. Compared with a static random-access memory (SRAM), the power consumption of DRAM is significantly high. It costs 640pj to access 32 bits of data from DRAM but costs only 5pj to access 8 Kb of data from SRAM (Horowitz, 2014). This study uses a learning system to store weights in synapses between neurons. The integration of storage and computation has become the key to solving speed and energy consumption problems. This learning system is implemented on a field-programmable gate array (FPGA), which has rich memory and computation resources (Wang et al., 2016; Chang and Culurciello, 2017; Yang et al., 2018, 2020).

While implementing an on-chip learning system on FPGA, the computation of global gradients requires a large number of state variables stored in registers, which is very luxurious for the FPGA. A series of algorithms based on surrogate-gradient are proposed to replace global gradients backpropagated layer by layer from outputs (Zenke and Ganguli, 2017; Sacramento et al., 2018; Neftci et al., 2019; Nøkland and Eidnes, 2019; Kaiser et al., 2020; Debat et al., 2021; Singanamalla and Lin, 2021). Although local gradients based on loss functions for each layer reduce the memory requirement, the memory complexity is still O(NT), where N is the number of nodes and T is the number of time steps in the time window (Kaiser et al., 2020). This study applies a multi-compartment leaky integrate-and-fire (MLIF) model to a SNN, which contains the basal dendrite, the apical dendrite, and the soma. The plateau potential in the apical dendrite guides the updates of synaptic weights. State variables are no longer required to be stored all the time to calculate gradients. The memory complexity is reduced to O(N). In order to match the digital circuits, shift multipliers (shift MUL) and piecewise linear (PWL) are applied to replace multiplication and complex nonlinear operations.

Further, we perform the system to classify spike patterns and reproduce their frequency distribution. With noise applied to the outputs of each soma, we also analyze the robustness and effectiveness of the system. The architecture of the learning system realized in this study consumes fewer resources and achieves high evaluation accuracy.

## Materials and methods

### The architecture of spiking neural network and multi-compartment leaky integrate-and-fire model

**FIGURE 1:**
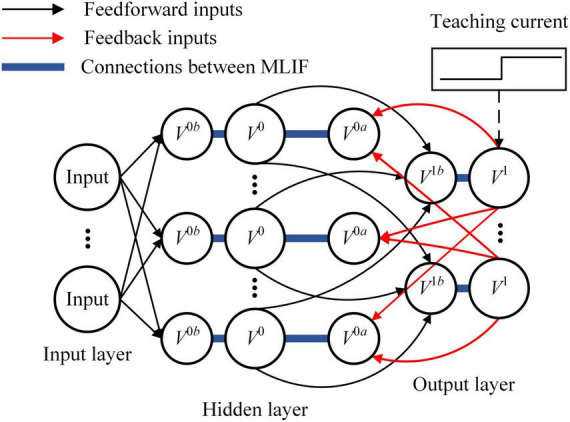
The architecture of spiking neural network (SNN) is depicted. There are three layers in the feedforward SNN. Input signals are encoded by the input layer and transmitted from the bottom to the top of SNN. An error is generated by the output layer with teaching current and backpropagated to the hidden layer with a dedicated channel different from the feedforward channel.

The SNN used in this study is divided into three layers, including the input layer, hidden layer, and output layer, as shown in Figure 1. Spike pattern inputs are encoded into spike trains by Poisson filters. The MLIF model in the hidden layer includes three compartments representing the basal dendrite, apical dendrite, and soma. The basal dendrite receives spike trains from the input layer, and the apical dendrite receives spike trains backpropagated from the output layer. Compared with the single-compartment LIF model, the MLIF model used in this study provides independent channels for information transmission. Dendritic current is transmitted to the soma and converted to the new spike trains. The somatic voltage of the MLIF model is described as follows:


(1) $$V^{0}\left(t+1\right)=V^{0}\left(t\right)+(g_{l}\left(V_{r\,e\,s}-V^{0}\left(t\right)\right)+g_{b}\left(V^{0\,b}\left(t\right)-V^{0}\left(t\right)\right)+g_{a}\left(V^{0\,a}\left(t\right)-V^{0}\left(t\right)\right))/C{}_{m},$$

where *V*^0^ is the somatic voltage of the MLIF model in the hidden layer, and *g*_*l*_ is the leak conductance. *C_m_* is the membrane capacitance, *g*_*a*_ and *g*_*b*_ are the conductance from apical dendrite to soma and from the basal dendrite to soma, *V*_*res*_ is the resting potential, *V*^0^*^b^* is the basal dendritic potential, and *V*^0^*^a^* is the apical dendritic potential. The basal and apical dendritic potentials are given by weighted sums of the filtered spike trains:


(2) $$V_{i}^{0\,b}\,\left(t\right)=\sum_{j}W_{i\,j}^{0}\,x_{j}\,\left(t\right)+b_{i}^{0},$$

(3) $$V_{i}^{0\,a}\,\left(t\right)=\sum_{j}Y_{i\,j}\,s_{j}^{1}\,\left(t\right),$$

(4) $$s\,\left(t\right)=\sum_{k}K\,\left(t-t_{k}\right),$$

(5) $$K\,\left(t\right)=\left(e^{-t/\tau_{l}}-e^{-t/\tau_{s}}\right)\,\Theta \,\left(t\right)/\left(\tau_{l}-\tau_{s}\right),$$

where *x* is the filtered spike trains from the input layer, *s*^1^ is the filtered spike trains from the output layer, *K*(*t*) is the kernel function, *W*^0^ is the feedforward synaptic weights from the input layer to the hidden layer, *Y* is the synaptic feedback weights from the output layer to the hidden layer, *b*^0^ is the bias of the MLIF model in the hidden layer, *t*_*k*_ is the *k*th spike time of the spike trains, *τ_*l*_* and *τ_*s*_* are long and short time constants, and Θ(*t*) is the Heaviside step function.

The firing rate of the MLIF model is described as follows:


(6) $$\phi^{0}\,\left(t\right)=\phi_{\max}\,\sigma \,\left(V^{0}\,\left(t\right)\right)=\phi_{\max}\,\frac{1}{1+e^{-V^{0}\,\left(t\right)}},$$

where ϕ^0^(*t*) is the firing rate of the MLIF model at time *t*, *ϕ_*max*_* is the maximum firing rate, and σ[*V*^0^(*t*)] is the sigmoid function as a nonlinear activation function. The Poisson filter samples spike trains based on the firing rate of the MLIF model.

The MLIF model in the output layer includes two compartments representing the basal dendrite and soma. A teaching current is added to the soma of the MLIF model in the output layer, which contains an excitatory current and an inhibitory current. The somatic voltage of the MLIF model in the output layer is described as follows:


(7) $$V^{1}\left(t+1\right)=V^{1}\left(t\right)+(g_{l}\left(V_{r\,e\,s}-V^{1}\left(t\right)\right)+g_{d}\left(V^{1\,b}-V^{1}\left(t\right)\right)+I\left(t\right))/C{}_{m},$$

(8) $$I\,\left(t\right)=g_{E}\,\left(E_{E}-V^{1}\,\left(t\right)\right)+g_{I}\,\left(E_{I}-V^{1}\,\left(t\right)\right),$$

(9) $$V_{i}^{1\,b}\,\left(t\right)=\sum_{j}W_{i\,j}^{1}\,s_{j}^{0}\,\left(t\right)+b_{i}^{1},$$

where *V*^1^ is the somatic voltage of the MLIF model in the output layer, *g*_*d*_ is the conductance from basal dendrite to soma, *V*^1^*^b^* is the basal dendritic potential, *I*(*t*) is the teaching current added to the MLIF model, *g*_*E*_ and *g*_*I*_ are the excitatory and inhibitory nudging conductance, *s*^0^ is the filtered spike trains from the hidden layer, *W*^1^ is the feedforward synaptic weights from the hidden layer to the output layer, and *b*^1^ is the bias of the output layer.

### Learning rules based on plateau potentials

Neuron models used in this study generate spikes based on the Poisson process. The training period consists of two phases: the forward phase and the target phase. Each phase continues 100 ms, and all synaptic weights and biases are updated at the end of the target phase. Notably, the synaptic feedback weights from the output layer to the hidden layer are fixed. In the forward phase, the teaching current *I* is kept at 0. In the target phase, *g*_*E*_ = 1 and *g*_*I*_ = 0 are applied to the output neuron according to the default label. The other neurons in the output layer are inhibited with *g*_*E*_ = 0 and *g*_*I*_ = 1. Synaptic weights and biases in the hidden layer are updated based on the plateau potentials of apical dendrites as follows:


(10) $$W^{0}=W^{0}-\eta^{0}\,P^{0}\,\frac{g_{b}}{g_{l}+g_{b}+g_{a}}\,\left(\alpha^{t}-\alpha^{f}\right)\,\phi_{\max}\,\sigma^{\prime }\,\left({\bar{V}}^{0\,f}\right)\,∙\,{\bar{x}}^{f},$$

(11) $$b^{0}=b^{0}-\eta^{0}\,P^{0}\,\frac{g_{b}}{g_{l}+g_{b}+g_{a}}\,\left(\alpha^{t}-\alpha^{f}\right)\,\phi_{\max}\,\sigma^{\prime }\,\left({\bar{V}}^{0\,f}\right),$$

(12) $$\sigma^{\prime }\,\left({\bar{V}}^{0\,f}\right)=\sigma \,\left({\bar{V}}^{0\,f}\right)\,\left(1-\sigma \,\left({\bar{V}}^{0\,f}\right)\right),$$

(13) $$\alpha^{f}=\sigma \,\left(\frac{1}{\Delta \,t}\,\int_{t_{1}-\Delta \,t}^{t_{1}}V^{0\,a}\,\left(t\right)\,dt\right),$$

(14) $$\alpha^{t}=\sigma \,\left(\frac{1}{\Delta \,t}\,\int_{t_{2}-\Delta \,t}^{t_{2}}V^{0\,a}\,\left(t\right)\,dt\right),$$

where η^0^ is the learning rate, *P*^0^ is the scaling factor of the hidden layer, α*^f^* and α*^t^* are plateau potentials in the forward phase and the target phase, and $\bar{V}$^0^*^f^* is the average somatic voltage of the MLIF model in the hidden layer during a forward phase, $\bar{x}$*^f^* is the average input from the input layer during the forward phase, σ’ is the derivative of the sigmoid function, “⋅” is the matrix multiplication, *t*_1_ and *t*_2_ are the duration of the forward phase and the target phase, and Δ*t* is the unstable time. Synaptic weights and biases in the output layer are updated based on the error between predictions of SNN and labels as follows:


(15) $$W^{1}=W^{1}-\eta^{1}\,P^{1}\,\frac{g_{d}}{g_{l}+g_{d}}\,\left({\bar{\phi }}^{1\,t}-{\bar{\phi }}^{1\,f}\right)\,\phi_{\max}\,\sigma^{\prime }\,\left({\bar{V}}^{1\,f}\right)\,∙\,{\bar{s}}^{0\,f},$$

(16) $$b^{1}=b^{1}-\eta^{1}\,P^{1}\,\frac{g_{d}}{g_{l}+g_{d}}\,\left({\bar{\phi }}^{1\,t}-{\bar{\phi }}^{1\,f}\right)\,\phi_{\max}\,\sigma^{\prime }\,\left({\bar{V}}^{1\,f}\right),$$

(17) $${\bar{\phi }}^{1\,f}=\phi_{\max}\,\sigma \,\left({\bar{V}}^{1\,f}\right),$$

(18) $${\bar{\phi }}^{t}=\phi_{\max}\,\sigma \,\left({\bar{V}}^{t}\right),$$

where η^1^ is the learning rate, *P*^1^ is the scaling factor of the output layer, $\bar{\phi }$^1^*^f^* and $\bar{\phi }$^1^*^t^* are the average firing rates of output neurons in the forward phase and target phase, and $\bar{s}$^0^*^f^* is the average filtered input from the hidden layer. We simulate a 784-500-10 SNN on a computer with MATLAB before implementing the learning system on the FPGA. The SNN is trained to classify the handwriting digits of the MNIST dataset and reaches an accuracy of 96.13% after 60 epochs. It suggests that the SNN learns satisfactorily based on the learning rule. The parameters used in this simulation are shown in Table 1 (Guerguiev et al., 2017).

**TABLE 1:** Parameter values of spiking neural network (SNN) (Guerguiev et al., 2017).

Parameters	Values	Parameters	Values
*dt*	1	*g*_*a*_	0
*φ_*max*_*	0.2	*g*_*b*_	0.6
*τ_*l*_*	10	*g*_*l*_	0.1
*τ_*s*_*	3	*V*_*res*_	0
*E*_*E*_	12	*C*_*m*_	1
*E*_*I*_	−12	Δ*t*	30
*g*_*d*_	0.6	η^0^, η^1^	0.01
*P* ^0^	20/*φ_*max*_*	*P* ^1^	20/*φ_*max*_*^2^

### Linear approximated function

**FIGURE 2:**
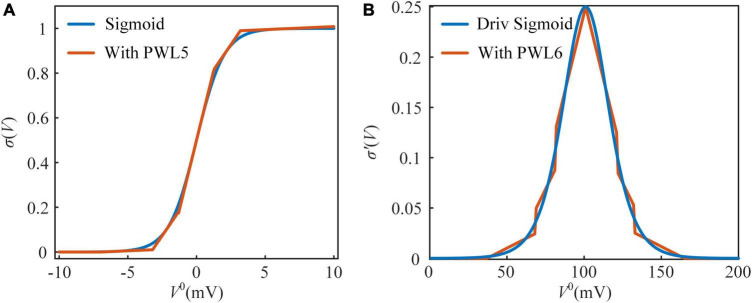
The approximated sigmoid functions and their derivative functions. **(A)** The sigmoid function and its approximated function with piecewise linear 5 (PWL5). **(B)** The derivative of the sigmoid function and its approximated function with piecewise linear 6 (PWL6).

The sigmoid function is used to convert the somatic potential to a spiking frequency. Due to the exponential and reciprocal operations in the sigmoid function, it is not friendly for implementation on hardware (Lian et al., 2019; Heidarpur et al., 2020). These operations are usually realized by a large number of adders and multipliers on the FPGA. Shift and addition are the easiest operations realized on the FPGA. However, multiplication usually consumes decades more resources than addition. In this study, piecewise linearization (PWL) is used to replace nonlinear functions with several linear functions. In Figure 2A, the sigmoid function is divided into five PWL segments with two additional limit rules (Soleimani et al., 2012; Hayati et al., 2015). Accordingly, the PWL5 model of the sigmoid function is computed by the following equation:


(19) $$\sigma_{l}\,\left(V\right)=\left\{ \begin{array}{ccc} m_{0}\,V+k_{0}, & V\leq N_{0} & \\ m_{1}\,V+k_{1}, & N_{0}<V\leq N_{1} & \\ m_{2}\,V+k_{2}, & N_{1}<V\leq N_{2}, & \\ m_{3}\,V+k_{3}, & N_{2}<V\leq N_{3} & \\ m_{4}\,V+k_{4}, & V>N_{3} & \end{array}\right.$$

where σ_*l*_ is the sigmoid function with PWL5, *m*_*i*_ and *k*_*i*_ are the slope and intercept of lines in PWL5, and *N*_*i*_ is the scope of the border. We exhaust *N*_*i*_, *m*_*i*_, and *k*_*i*_ to minimize the root-mean-square error (RMSE), which is computed by the following equation:


(20) $$R\,M\,S\,E=\sqrt{\frac{1}{N}\,\sum_{i=1}^{N}{\left(X_{s\,o\,f}\,\left(i\right)-X_{h\,a\,r}\,\left(i\right)\right)}^{2}},$$

where *N* is the number of values in exhaustion, the steps of *N*_*i*_, *m*_*i*_, and *k*_*i*_ are 0.1, 0.01, and 0.01 in exhaustion. The sigmoid function with PWL5 is clipped to 0–1. In Figure 2B, the derivative of the sigmoid function is divided into six PWL segments, and it with PWL6 is clipped to 0–∞. The evaluation results of Sigmoid and their derivative functions are shown in Table 2. The evaluation criteria include RMSE, mean absolute error (MAE), and R-square (R^2^). MAE and R^2^ are computed as follows:


(21) $$M\,A\,E=\frac{1}{N}\,\sum_{i=1}^{N}\left|X_{s\,o\,f}\,\left(i\right)-X_{h\,a\,r}\,\left(i\right)\right|,$$

(22) $$R^{2}=1-\frac{\sum_{I=1}^{N}{\left(X_{s\,o\,f}\,\left(i\right)-X_{h\,a\,r}\,\left(i\right)\right)}^{2}}{\sum_{I=1}^{N}{\left({\bar{X}}_{s\,o\,f}-X_{s\,o\,f}\,\left(i\right)\right)}^{2}},$$

where *X*_*sof*_(*i*) and *X*_*har*_(*i*) are the results of simulation on software and implementation on FPGA at the *i*th iteration, *N* is the total number of iterations, and $\bar{X}$_*sof*_ is the mean of *X*_*sof*_(*i*). The results of error evaluation with three indicators are shown in Table 3. With PWL models, only shift and addition operations are needed while calculating nonlinear functions.

**TABLE 2:** Coefficients of approximated functions.

Coefficients	Approximated function	
	Sigmoid	Derivative of sigmoid
(*m*_0_, *k*_0_, *N*_0_)	(0.0078125, 0.05,3.4)	(0.0078125, 0.05,3.2)
(*m*_1_, *k*_1_, *N*_1_)	(0.0625, 0.24,1.3)	(0.03125, 0.15,2)
(*m*_2_, *k*_2_, *N*_2_)	(0.25, 0.5, 1.3)	(0.0625, 0.25, 0)
(*m*_3_, *k*_3_, *N*_3_)	(0.0625, 0.76, 3.4)	(0.0625, 0.26, 2)
(*m*_4_, *k*_4_, *N*_4_)	(0.0078125, 0.95,−)	(0.03125, 0.15, 3.2)
(*m*_5_, *k*_5_, *N*_5_)	(−,−,−)	(0.0078125, 0.05,3.2)

**TABLE 3:** Error evaluation results.

	RMSE	MAE	R^2^
Sigmoid	0.0101	0.0049	0.9995
Derivative of Sigmoid	0.0015	2.1643 × 10^–4^	0.9948

### Neuromorphic system architecture overview

The SNN used in this study comprises four parts: inputs, MLIF models, outputs, and synaptic plasticity modules. The learning system is implemented on the Altera Stratix V Advanced Systems Development Kit with Altera Stratix V 5SGXEA7N2F45C2N FPGA, which is available at https://github.com/TianGaoTJU/Learning-System-of-Spiking-Neural-Network-with-Multi-compartment-LIF-Neurons. This learning system on the FPGA is used to classify spike patterns and reproduce frequency distribution. To keep a balance between the accuracy and resource consumption of calculation, we used 24-bit fixed-point data for calculation. The value of data is computed as follows:


(23) $${\left(-1\right)}^{s\,i\,g\,n}\times \left(i\,n\,t\,e\,g\,e\,r+f\,r\,a\,c\,t\,i\,o\,n/2^{16}\right),$$

The 0–15th bits are the fraction part, the 16–22nd bits are integral, and the 23rd is the significant part. If the sign part is 0, the data is positive. If the sign part is one, it is negative.

**FIGURE 3:**
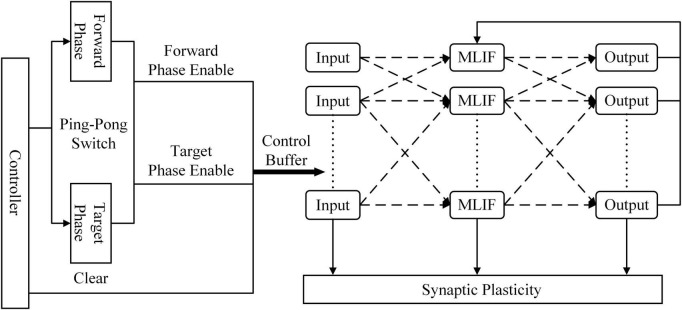
Overview of the spiking neural network (SNN) architecture implemented on the field-programmable gate array (FPGA). The controller is used as a system clock counter and enables signals. The ping-pong switch controls enable signals for the forward phase and target phase.

Figure 3 shows the architecture of the learning system implemented on the FPGA. The controller contains a counter used as a system clock. The counter increases at the posedge of the system clock from 0 to 200. When the counter reaches 100, the ping-pong switch is sloped to the target side. When the counter reaches 200, the ping-pong switch is sloped to the forward side. At the beginning of the forward phase, a clear signal is transmitted to all modules to reset the SNN to its initial state. There is a clear port, a forward phase enable port, and a target phase enable port in the synaptic plasticity module. In the forward phase, the forward phase enables signal = 1, and the target phase enables signal = 0. In the target phase, the settings are the opposite. More details of control signals are shown in Figure 6. The outputs of SNN are spike trains. Each output neuron has a dedicated counter that counts the soma spikes. The location of the output neuron with the most spikes is considered the prediction of SNN.

### Implementation of the multi-compartment leaky integrate-and-fire model

**FIGURE 4:**
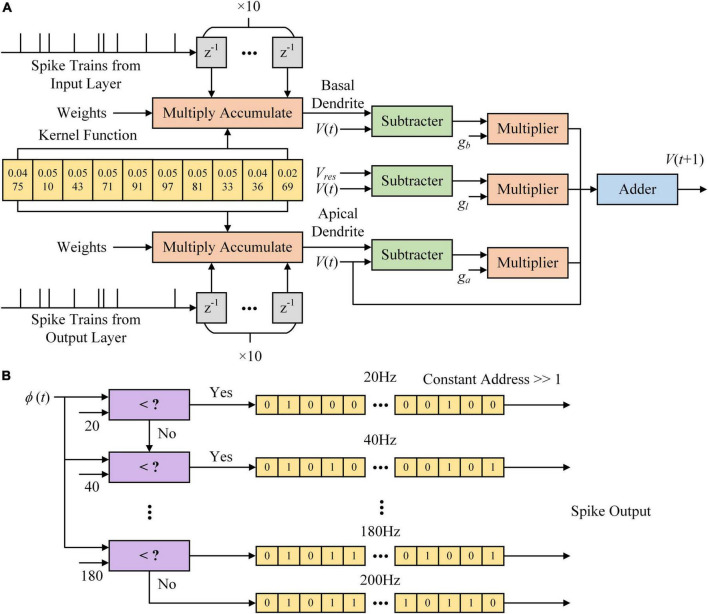
Multi-compartment leaky integrate-and-fire (MLIF) model implemented on the field-programmable gate array (FPGA). **(A)** The architecture of somatic voltage used in the MLIF model. **(B)** The architecture of the Poisson process is used to generate spike trains based on the firing rate of the MLIF model. Spike trains with different firing rates are stored in memory. When a spike output is read out, the constant address is shifted right a bit.

Figure 4 shows the architecture of the MLIF model in the hidden layer. Figure 4A shows that the kernel function is calculated in a 10 ms time window. K (t) results in ten timesteps stored in shift registers shown as yellow blocks. Spike trains from the input layer are delayed from 1 to 10 ms and multiplied with ten *K* (t) values stored in registers. The *z*^–1^ block in gray is a register with a single bit and stores the spike at the latest timestep. *K* (t) values are multiplied by synaptic weights and accumulated in the Multiply Accumulate (MAC) module. The dendritic and somatic potential differences are multiplied by the conductance. The new somatic potential is calculated based on the sum of dendritic currents and leakage currents. The MLIF model in the output layer differs from that in the hidden layer, which only consists of the basal and somatic compartments.

**FIGURE 5:**
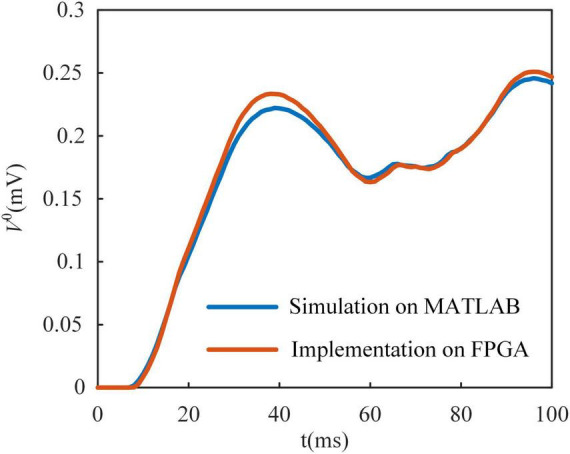
Multi-compartment leaky integrate-and-fire (MLIF) model implemented on field-programmable gate array (FPGA) is compared with it simulated in MATLAB with the same random input. There is only a small difference between the MLIF model in simulation and its implementation on the FPGA.

In Figure 4B, we show the Poisson filter used to sample spikes from the firing rate of the MLIF model. The firing rate of each model is clipped to 0–200 Hz and distributed over ten frequency segments. Each frequency segment covers a range of 20 Hz. We use the random function in MATLAB to generate these ten Poisson spike trains on a computer. The parameter λ of the function is set as 0.02, 0.04, …, 0.2, where λ is the average incidence of random events happening per millisecond. The spike trains are clipped to 0–1. We used the chi-square test to evaluate these Poisson spike trains. With a significance level of 0.05, these spike trains conform to the Poisson distribution. Then these spike trains are stored in shift registers as the yellow blocks in Figure 4B. The shift register outputs a bit of data at the constant address according to ϕ(t), and then the constant address is shifted right a bit. Figure 5 shows the somatic potential of the MLIF model implemented on FPGA and simulated in MATLAB on the computer with the same input. The results of the error evaluation of the MLIF model are as follows, i.e., RSME = 0.0057, MAE = 0.0042, and R^2^ = 0.9944.

**FIGURE 6:**
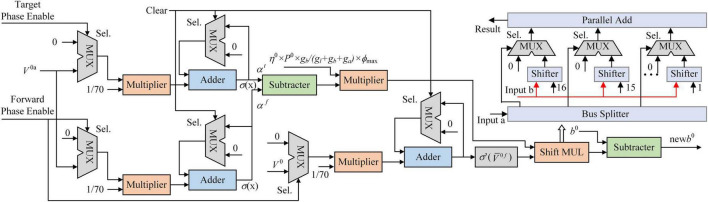
The architecture of synaptic plasticity. The synaptic plasticity module is implemented on the field-programmable gate array (FPGA) as an on-chip learning system. A shift multiplier (MUL) module is proposed to replace DSP blocks on the FPGA to reduce logical resource consumption.

### Implementation of synaptic plasticity

There are three input ports in the synaptic plasticity module implemented on FPGA: the forward phase enable, the target phase enable, and clear. In the controller, the forward phase enable and the target phase enable are accessed with a ping-pong switch. When the forward phase enable is set to 1, the target phase enable is set to 0. Figure 6 shows the architecture of the synaptic plasticity module for *b*^0^. The MUX block is a selector with three input ports and an output port. When the Sel. port is 0, the MUX block outputs the data on the first input port. When the Sel. port is 1, the MUX block outputs the data on the second input port. In the forward phase (without the first 30 ms), *V*^0^*^a^* is transmitted into the multiplier and decreased to 1/70 of its original value. The sigmoid function with PWL5 converts the average potential of the apical dendrite to the firing rate in the forward phase. Due to the target phase enable being set to 0, the inputs of *α^t^* are clamped at 0. In the target phase (without the first 30 ms), the target phase enable is set to 1, and the forward phase enable is set to 0. The input to *α^f^* is clamped at 0, keeping the value. *V*^0^*^f^* is calculated in the same way.

Two kinds of multipliers are implemented in the synaptic plasticity module. One is used for the multiplication of a constant and a variable. This multiplier is composed of shifters and adders. Another is the Shift MUL module, which multiplies two variables (Yang et al., 2021). The “Input a” and “Input b” are two variable inputs of the Shift MUL module. Note that the value of “Input a” is expected to be between 0 and 1 for the exact calculation, and thus the 16–23rd bits of “input a” are dropped, and the 0–15th bits are split into 16 single bits in a bus splitter. The outputs of the bus splitter are numbered from 16 to 1 according to the importance of each output port, from the least significant to the most significant bit. The “Input b” is shifted right in the shifter block according to the input number from 16 to 1. The MUX block is used to choose the input flow. The number 0 is added to the first data line of MUX. And the variable “Input b” is added to the second data line after being shifted right by the barrel shifter block. Outputs of the MUX blocks are added in a parallel adder. The sum of MUX blocks is considered the multiplication result. The same indicators are used to evaluate the errors of Shift MUL. RSME is 1.0703 × 10^–4^, MAE is 9.8480 × 10^–5^, and R^2^ is 1.0000.

We also compare the compilation results of the shift MUL module and the multiplier summarized in Table 4. The multiplier module is an IP block provided by Quartus and DSP Builder software, which consumes 436 LUTs. A shift MUL module consumes 185 LUTs and 185 registers. One LUT and one register are integrated into a logic element (LE) of an FPGA. This result indicates that the Shift MUL module only requires 185/436 LEs of the multiplier module, suggesting high resource utilization efficiency.

**TABLE 4:** Resource utilization of multipliers.

	LUT	Register/FF
Shift MUL	185	185
Multiplier	436	0

## Results

**FIGURE 7:**
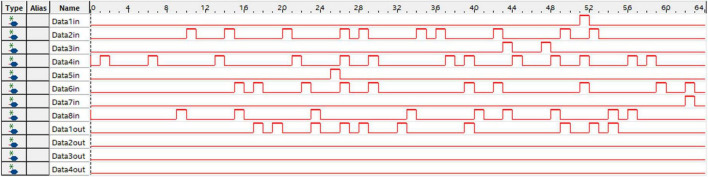
An example of spike pattern inputs and outputs of spiking neural network (SNN) on the field-programmable gate array (FPGA). Spike trains with a certain frequency distribution are input to SNN for classification. The output port “Data1out” outputs the most spikes, reflecting the prediction label according to this spike pattern.

An 8 × 10 × 4 SNN (with 120 synapses) is implemented on the FPGA to evaluate the learning system. Four spike patterns with different frequency distributions are input to the SNN. In these spike patterns, the maximum spike frequency is 200 Hz, and the minimum spike frequency is 20 Hz. Figure 7 shows the inputs and outputs of the SNN on the FPGA with the SignalTap II Logic Analyzer. Spike trains in 200 Hz are input to “Data2in,” “Data4in,” “Data6in,” “Data8in,” and spike trains in 20 Hz are input to “Data1in,” “Data3in,” “Data5in,” and “Data7in.” Output ports “Data1out”–“Data4out” are spike trains output by MLIF models in the output layer. It can be found that the first neuron outputs the most spikes, so the prediction label of the SNN is 1. In this experiment, SNN realizes a high accuracy of 100.0% after 500 epochs. The resource utilization and power consumption are shown in Table 5. Compared with the other two previous works, our SNN performs higher Fmax, fewer look-up tables (LUTs), and less power consumption. The total power dissipation is 3.644 watts, as shown in Table 4. The core dynamic power dissipation is 2.2215 watts, and the static power dissipation is 989.08 mW. The I/O power dissipation is 433.44 mW. A 2 × 2 × l SNN with six synapses is implemented on FPGA based on the on-chip back-propagation learning algorithm designed by Vo (2017). Further, Mazouz and Bridges (2021) implement an 8 × 8 CNN based on a back-propagation learning algorithm. Table 5 shows the compilation results of neural networks implemented on FPGA, which are extended to 120 synaptic connections. The SNN implemented by Vo (2017) requires 5.6 times the logic elements of our implementation. The utilization of the CNN is 1.6 times that of the SNN in this study if it is extended to 120 neurons. These results suggest that our SNN performs more efficiently in resource utilization.

**TABLE 5:** Resource utilization and comparison with previous works.

	LUT	Register/FF	Power	Fmax	Device
This study	196469	197154	3.644 W	216.54 MHz	Stratix V
Vo (2017)	1103160	584640	−	−	Spartan 3E
Mazouz and Bridges (2021)	274125	−	4.982 W	200 MHz	Zynq-7100

**FIGURE 8:**
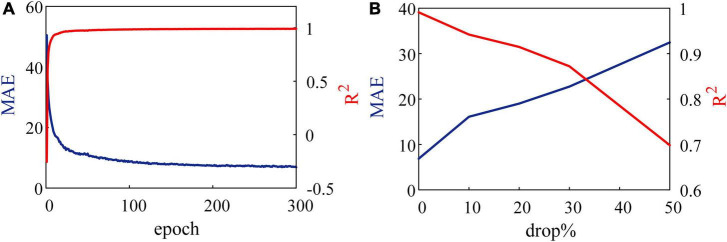
Results of reproducing spike patterns. **(A)** Without noise, spiking neural network (SNN) reproduces input patterns accurately. **(B)** Noise in the form of dropping with a constant probability is added to an SNN.

Another experiment is tested on the 8 × 10 × 8 SNN (with 160 synapses) on the FPGA. In this experiment, SNN is expected to reproduce the patterns with the specified frequency distribution (Mohemmed and Schliebs, 2012). In Figure 8, errors between random pattern input and frequency distribution output by SNN are evaluated in MAE and R2. Figure 8A shows the results of inputs without any noise. At the end of each training epoch, the SNN is tested and outputs spikes. Trained after 300 epochs, MAE and R^2^ are 6.8904 and 0.9910. When compared with the range of 200 Hz, the SNN reproduces the input spike pattern accurately.

Further, we add a drop noise to spike trains and show the results trained after 300 epochs in Figure 8B, where the abscissa is the probability of dropping. When 50% of input spikes are decreased by noise, MAE increases to 32.46, and R^2^ reduces to 69.55%. These results suggest that the learning system performs well under robustness to noise.

## Discussion

This study applies dendrite-guided synaptic plasticity to the neuromorphic learning system. In contrast to the gradients backpropagated from top-down in ANNs, two dendrites in the MLIF model receive spike trains as inputs. Dendrites convert the input frequency into the potential. After a short oscillating period, the potential in dendrites reaches a plateau. If potentials in dendrites are at a high level, the presynaptic neuron fires at a high frequency. While training the SNN, the teaching current is added to neurons in the output layer. It could be transmitted to the apical dendrite, which guides the behavior of neurons in a lower layer. The gradients are not backpropagated from the output layer but calculated by the presynaptic activities and potentials in the dendrites. Benefiting from the reduction in memory complexity and synaptic plasticity, data used for learning could be stored on FPGA, not off-chip memory. It further contributes to the decrease in energy consumption in dataflow between on-chip and off-chip memories, which is considered a challenge in energy and speed. In order to match the learning system to the digital circuits, PWL and Shift MUL are applied to optimize nonlinear functions and multiplication between two fixed-point data.

As a computing unit independent of the soma, the dendrite enriches the possibility of a learning mechanism in the system. It has been proven that the prediction error between dendrites and soma can be used for synaptic plasticity. Urbanczik and Senn (2014) minimize the discrepancies between somatic firings and local dendritic potential to train their SNN. The error is generated continuously and drives synapses to be constantly updated. Sacramento et al. (2018) use the error between predictive activities from lateral interneurons and feedback activities to train the SNN. Their model does not need separate phases, and synaptic plasticity is driven by the local dendritic prediction errors continuously in time. These algorithms proved excellent and provided ways to solve credit assignment problems. However, continuous learning during training drives the synaptic plasticity module on the FPGA to work all the time at high speed. The lateral synaptic connections also increase the number of synaptic plasticity modules on FPGA. Although these two learning rules achieve extremely high accuracy in testing, we need a hardware-friendly learning rule to implement the SNN on FPGA. We divide the training period into two phases. Synaptic weights are updated at the end of the target phase. Only the plateau potentials in the apical dendrites are required to guide the updates of synaptic weights. The plateau potential is obtained by accumulating the potential in the apical dendrite, which consumes a fixed-point adder and two 24-bit registers. The power consumption of the learning system on FPGA is mainly generated by the transmission of spikes between neurons and the update of synaptic plasticity modules. We use the discrepancies between the plateau potentials of the apical dendrites in two phases to train the SNN, and weights are updated only once at the end of the target phase. The FPGA works at an extremely high speed compared to the CPU, but the memory and logical resources of the FPGA are scarce. Thus, high-efficient utilization of resources on the FPGA is the most important. Besides, while realizing the same learning system, the power consumption of FPGA is usually a few tenths of that of a GPU. Therefore, power consumption is usually considered the second most important standard for FPGA. We aim to realize a learning system with low power consumption and high resource utilization on FPGA. The SNN and architecture of implementation in this study are best suitable for this goal.

One of the main energy consumptions of the learning system occurs in data transmission. Spiking neurons generate spike trains, which only include 0 or 1 signal. Meanwhile, SNN transmits signals at 0 or 1 instead of continuous membrane potential. These 1-bit signals greatly reduce the requirements for bandwidth and energy during data transmission. After the dendrite of the MLIF model receives spike trains, the postsynaptic potential is triggered by a flip-flop (FF) without MAC operation. It has been shown that SNN works on FF and simple operations, not LUTs (Pei et al., 2019). In particular, the MLIF model used in this study generates spikes with a certain probability based on its membrane potential. As a result, the membrane potential is stored with less precision, and the neuromorphic system presents better robustness, as shown in Figure 8. Although neuromorphic learning systems are still being studies and have many limitations, embedded devices will present advantages in a broad category of applications with such neuromorphic learning systems.

## Conclusion

In this study, a high-efficient neuromorphic learning system based on MLIF models is realized on a FPGA. We use the discrepancies between the plateau potentials in two phases to train the SNN. The synaptic weights are updated only once at the end of the target phase, which facilitates FPGA implementation with fewer memory resources and energy. The shift MUL module and PWL mechanism are applied instead of multiplication and complex nonlinear operations. The neuromorphic learning system is implemented on the Stratix V FPGA. For important units and modules in this learning system, error evaluation is applied based on computer simulations and hardware experiments. Results of resource utilization and performance in two tasks support the notion that the neuromorphic learning system works more efficiently.

## Data availability statement

The original contributions presented in this study are included in the article/supplementary material, further inquiries can be directed to the corresponding author.

## Author contributions

All authors listed have made a substantial, direct, and intellectual contribution to the work, and approved it for publication.
